# 

**Published:** 1908-11

**Authors:** 


### BOOKS RECEIVED.

A Manual of Diseases of the Nose and Throat. By Cornelius G. Coakley, M.D., Clinical Professor of Laryngology in the University and Bellevue Hospital Medical College, New York. New (4th) edition, 12mo, 604 pages, with 126 engravings and 7 colored plates. Cloth, $2.75 net. Lea & Febiger, Publishers, Philadelphia and New York, 1908.

Pathogenic Microörganisms, including Bacteria and Protozoa. A practical manual for students, physicians and health officers. By William H. Park, M.D., Professor of Bacteriology and Hygiene in the University and Bellevue Hospital Medical College, New York. New (third) edition, thoroughly revised and much enlarged. Octavo,

648 pages, with 176 illustrations and 5 full-page plates. (Cloth, $3.75, net. Lea & Febiger, Philadelphia and New York, 1908.

High-Frequency Currents by Frederick Finch Strong, M.D. Instructor in Electro-therapeutics at Tuft's College Medical School, Boston. With 183 illustrations in the text. Rebman Company, 1123 Broadway, New York.

Textbook of Nervous Diseases and Psychiatry for the use of students and practitioners of medicine, by Charles L. Dana, A.M., M.D., LL.D., professor of nervous diseases in Cornell University; neurologist to the Montefiore Hospital; neurologist to the Woman's Hospital, etc. Seventh edition. Two hundred and sixty-one engravings; three plates in black and colors, pp. 782. New York: William Wood & Company. 1908. (Price, cloth $5.00 net.)

Operative Midwifery, by J. M. Munro Kerr, M.B., C. M. Glas. Fellow of the faculty of physicians and surgeons, Glasgow; obstetric physician to Glasgow Maternity Hospital; gynecologist to the Western Infirmary, etc., with 294 illustrations in the text. New York: William Wood & Company. 1908. (Price, $6.50 net. Cloth.)

Textbook of Operative Surgery, covering the surgical anatomy and operative technic involved in the operations of general surgery designed for practitioners and students, by Warren Stone Bickham, M.D., Phar.M., junior surgeon to Touro Hospital, New Orleans etc. Third edition, greatly enlarged. 854 illustrations, pp. 1206. W. B. Saunders Company, Philadelphia and London. 1908. (Price, cloth $6.50 net.)

Manual of Clinical Diagnosis by James Campbell Todd, Ph.B., M.D., associate professor of pathology Denver and Gross College of Medicine, University of Denver, etc. Illustrated. W. B. Saunders Company, Philadelphia and London. 1908. (Price, $2.00 net.)

The Campaign against Tuberculosis in the United States, including directory of institutions dealing with tuberculosis in the United States and Canada. Compiled under the direction of the National Association for the Study and Prevention of Tuberculosis by Philip P. Jacobs, New York: Charities Publication Committee 1908. (Price, $1.00 net.)

On the Means of the Prolongation of Life, by Sir Hermann Weber, M.D., F.R., C.P. Consulting physician to the German Hospital, the National Hospital for consumption, Ventnor, the Mount Vernon Hospital for consumption and a member of the consulting committee of King Edward VII sanatorium at Midhurst, London. John Bale, Sons, and Danielsson, L't'd., Oxford House 83-91 Great Titchfield street, Oxford street, West. 1908. (Price, $1.25 net.)

Spectacles and eyeglasses, their forms, mounting, and proper adjustment, by R. J. Phillips, M.D., ophthalmologist to Presbyterian Orphanage, etc. Fourth edition, revised, with 56 illustrations. Philadelphia: P. Blakiston's Son & Company, 1012 Walnut street. 1908. (Price, $1.00 net.)

Transactions of the Thirtieth Annual Meeting of the American Laryngological Association held at Montreal, Canada, May 11, 12, 13, 1908. New York: Published by the association. The McConnell Printing Company, printers. 1908.

Intestinal Autointoxication, by A. Combe, M.D., professor of clinical pediatry at the University of Lausanne (Switzerland), together with an appendix on the lactic ferments with particular reference to their application to intestinal therapeutics, by Albert Fournier, formerly demonstrator at la Sorbonne, Paris. Only authorised English adaptation by William Gaynor States, M.D., clinical assistant in rectal and intestinal diseases at the New York Polyclinic. Eighteen figures, four colored. Rebman Company, New York. 1908. (Price, cloth $4.00.)

Refraction of the Eye, by Harry C. Parker, M.D., Clinical Professor of Ophthalmology, Indiana University School of Medicine, Indianapolis. With 106 illustrations. Philadelphia and London: W. B. Saunders Company. 1908. (Price, $1.25 net.)

Reference Handbook for Nurses by Amanda K. Beck, Graduate of the Illinois Training School for Nurses. Second edition. Revised. Philadelphia and London. W. B. Saunders Company. 1908. (Price, $1.25 net.)

Obstetric and Gynecologic Nursing by Edward P. Davis, A.M., M.D, Professor of Obstetrics in the Jefferson Medical College, Philadelphia; Obstetrician and Gynecologist to the Philadelphia Hospital; Consultant to the Preston Retreat. Third edition, thoroughly revised. Philadelphia and London: W. B. Saunders Company. 1908. (Price, $1.75 net.)

Obstetrics for Nurses. By Joseph B. DeLee, M.D., Professor of Obstetrics in the Northwestern University Medical School, Chicago. Third Revised Edition. 12mo, of 512 pages, fully illustrated. Philadelphia and London: W. B. Saunders Company. 1908. (Cloth, $2.50 net.)

Textbook of General Bacteriology. By Edwin O. Jordan, Ph.D. Professor of Bacteriology in the University of Chicago and in Rush Medical College. Fully illustrated. Philadelphia and London: W. B. Saunders Company. 1908. (Price, $3.00 net.)

Textbook of Diseases of Women. By Charles B. Penrose, M.D., Ph.D., formerly Professor of Gynecology in the University of Pennsylvania; Surgeon to the Gynecean Hospital, Philadelphia. With 225 illustrations. Sixth edition revised. Philadelphia and London: W. B. Saunders Company. 1908. (Price, cloth $3.75; half-morocco, $5.25 net.)

---

"Doctor," said the shrewd-looking man, "how many feet of gas does it take to kill a man?"

"That's rather a queer question," replied the doctor. "Why do you wish to know?"

"Well, you see, one of the guests at my hotel used enough of it to kill himself, and I want to send in a proper bill to his executors."—*Philadelphia Press.*

---

"Here is a doctor who says you mustn't eat when you're worried."

"But suppose you're always worried for fear you ain't going to get anything to eat?"—*Cleveland Plain Dealer.*

---

Lobster and champagne for supper—that's high jinks. Sawdust and near-coffee for breakfast—that's hygiene. Between these two eminences, however, there's room for some genuine living.—*Life.*

